# Defining an optimal control for RNAi experiments with adult *Schistosoma mansoni*

**DOI:** 10.1038/s41598-023-36826-6

**Published:** 2023-06-16

**Authors:** Max F. Moescheid, Oliver Puckelwaldt, Mandy Beutler, Simone Haeberlein, Christoph G. Grevelding

**Affiliations:** Institute of Parasitology, Biomedical Research Center Seltersberg (BFS), Justus Liebig University Giessen, Giessen, Germany

**Keywords:** Zoology, Biological techniques, Gene expression analysis, Genetic techniques, Computational biology and bioinformatics, Data mining, Molecular biology, RNAi, Transcriptomics

## Abstract

In parasites such as *Schistosoma mansoni*, gene knockdown by RNA interference (RNAi) has become an indispensable tool for functional gene characterization. To distinguish target-specific RNAi effects versus off-target effects, controls are essential. To date, however, there is still no general agreement about suitable RNAi controls, which limits the comparability between studies. To address this point, we investigated three selected dsRNAs for their suitability as RNAi controls in experiments with adult *S.* *mansoni *in vitro. Two dsRNAs were of bacterial origin, the neomycin resistance gene (*neoR*) and the ampicillin resistance gene (*ampR*). The third one, the green fluorescent protein gene (*gfp*), originated from jellyfish. Following dsRNA application, we analyzed physiological parameters like pairing stability, motility, and egg production as well as morphological integrity. Furthermore, using RT-qPCR we evaluated the potential of the used dsRNAs to influence transcript patterns of off-target genes, which had been predicted by si-Fi (siRNA-Finder). At the physiological and morphological levels, we observed no obvious changes in the dsRNA treatment groups compared to an untreated control. However, we detected remarkable differences at the transcript level of gene expression. Amongst the three tested candidates, we suggest dsRNA of the *E. coli ampR* gene as the most suitable RNAi control.

## Introduction

RNA interference (RNAi) is a highly conserved cellular mechanism used by diverse organisms to control foreign gene expression and chromosome function^1–5^. RNAi is induced by double-stranded RNA (dsRNA), which is processed by the RNAses Dicer and Drosha into 19–23 nt long, short interfering RNAs (siRNA). Together with argonaute proteins, these siRNAs form the RNA-induced silencing complex (RISC), which binds to specific RNA targets to mediate their degradation. Although initially discovered in the nematode *Caenorhabditis elegans*^6^, genes coding for components of the RNAi machinery are present in almost all eukaryotes with few exceptions^7^. RNAi has become a favorite tool in parasite research to study gene function, taking advantage of the organisms’ own RNAi machineries. This technique is particularly important for the field of parasitology since for many parasites, unlike model organisms, gene knock-out techniques have not been established yet^8^. In the parasitic flatworm *S. mansoni*, first approaches utilizing RNAi were reported in 2003^9,10^. Since then, RNAi has become an invaluable tool in reverse genetics for this and other parasites^5,8,11,12^.

For RNAi experiments, it is important but also difficult to standardize experimental approaches to aid reproducibility, interpretations, and conclusions due to the enormous variations in parasite biology and experimental set-ups in vitro and in vivo^13^. These include diverse strategies of introducing endogenous dsRNA by injection, electroporation, soaking, and other methods to silence target-gene expression at the transcriptional level. On top, the selection of target genes, readout strategies, and controls have to be carefully conceived^8^. Especially the choice of appropriate controls can significantly influence the interpretation of the outcome of an experiment. To this end, using non-homologous sequences has become a common practice for evaluating non-specific dsRNA effects. However, are these non-homologous sequences as suitable as commonly assumed against the background that they are chopped into 19–23 nt long siRNA pieces, which may accidentally find unwanted target sequences? The possibility that dsRNAs can exert off-target effects has been demonstrated in different biological systems^14^ (like *C. elegans*^15^, mammalian cells^16^ and *D. melanogaster*^17^), including schistosomes and other parasites (*S. mansoni*^18^, *S. japonicum*^19^, *Ascaris suum*^20^ and *Fasciola gigantia*^21^). In addition, treatment with high concentrations of dsRNA poses the danger of saturation of the RISC complex, which might lead to systemic dysregulation of gene expression^22–24^. Among the non-homologous sequences commonly used as controls in schistosome RNAi experiments are “scrambled” sequences of the actual target-oriented dsRNA, or non-schistosome dsRNAs targeting genes such as *gfp*, *mcherry* and *luciferase* (non-target dsRNAs). At first glance, these sequences are expected to cause no nonspecific effects. However, problems with these controls were reported like the altered expression of off-target genes^23,25^. This indicates the possibility that siRNAs were generated matching non-homologous sequences. To overcome such obstacles in schistosomes, few studies have addressed optimal settings for RNAi. However, these studies mainly focused on the juvenile schistosomulum stage^18,26^ and not on the adult parasites.

The aim of our study was to reassess commonly used non-target dsRNAs for their suitability as RNAi controls. To this end, we focused on adult *S. mansoni* that were cultured in vitro for three weeks. To study physiological, morphological, and molecular aspects, parameters like pairing stability of the male and female parasite, motility, and egg laying were monitored by bright-field microscopy, and worm morphology was assessed by confocal laser scanning microscopy (CLSM). In order to predict potential off-target genes, for which transcript levels might be affected, we used the software si-Fi (siRNA-Finder)^27^. This tool splits a dsRNA sequence in silico into x-mers, and subsequently maps potential target genes by sequence similarity using the BOWTIE algorithm. Additionally, si-Fi predicts efficient siRNAs by taking structural accessibility of the target site into account. The obtained results indicated the suitability of all three tested non-target dsRNAs if physiological and morphological parameters are matter of research. With regard to off-target effects at the transcriptional level, however, one of the tested dsRNAs, designed against *ampR*, outmatched the other dsRNAs. Our results provide a guideline for the best possible experimental design of RNAi in *S. mansoni* and a template for similar approaches in other organisms, for which RNAi controls are needed.

## Material and methods

### Parasite stock

A Liberian strain of *S. mansoni* was maintained in snails of the species *Biomphalaria glabrata* as intermediate host and in Syrian hamsters (*Mesocricetus auratus*) as definitive host^28^. Adult worms were obtained by hepatoportal perfusion at 42–49 days (d) post infection.

### Cloning of constructs for dsRNA synthesis

For dsRNA synthesis, T7 promoter-driven constructs were cloned based on the pJC53.2 vector backbone (kindly provided by Jim Collins, University Texas, Southwestern Medical Center). To prepare the vector for cloning, 1 µg of pJC53.2 plasmid DNA was digested by *Ahd*I (NEB) in a total volume of 50 µL of 1 × CutSmart buffer (NEB) at 37 °C for 2 h. The resulting DNA fragments were separated by agarose gel electrophoresis. Appropriate DNA bands were extracted using the Monarch DNA Cleanup and Gel Extraction Kit (NEB).

Amplicons of the selected target DNAs were generated by PCR using gene-specific primers. Fragments of about 500 bp length were generated from the *ampR* gene of pJC53.2 (primers: Ampicillin_fw 5′-GAG TAT TCA ACA TTT CCG TGT CGC-3′, Ampicillin_rev 5′-CGG TTC CCA ACG ATC AAG GC-3′) or the GFP (green fluorescent protein) sequence from pBluescript Hsp70-GFP-Hsp7029 (primers: GFP_fw 5′-GCA ACA TAC GGA AAA CTT ACC C-3′, GFP_rev 5′-CGT TGG GAT CTT TCG AAA GGG-3′). The *neoR* gene of pJC53.2 served as template for dsRNA synthesis as described below (2.4.). PCR amplification was performed using 1 µM of the respective primers and Q5 High-Fidelity Polymerase (NEB) according to recommended concentration. Template DNA was initially denatured at 98 °C for 3 min, followed by 35 amplification cycles, which consisted of denaturation at 95 °C for 30 s, primer annealing at 58 °C for 30 s and elongation at 72 °C for 0.45 min (BioRad, S1000 Thermal Cycler). Aliquots of generated PCR products were analyzed by agarose gel electrophoresis, cleaned using Monarch DNA Cleanup (NEB), extracted by the Gel Extraction Kit (Firma), and finally eluted in 20 µL elution buffer. This step was followed by an additional PCR to generate 3′A-overhangs (AccuPrime Taq DNA Polymerase High Fidelity kit; Invitrogen). For this purpose, 20 µL of the fragment-containing DNA eluate served as template, and primers for the corresponding template were used. Template DNA was initially denatured at 98 °C for 3 min, followed by five amplification cycles to generate 3′A-overhangs with the following cycling profile: denaturation at 95 °C for 30 s, primer annealing at 58 °C for 30 s, and elongation at 67 °C for 5 min (BioRad, S1000 Thermal Cycler). The resulting fragments were cleaned up as described before.

Afterwards, the fragments were ligated into pJC53.2 using T4 ligase (NEB) as described in the manufacturer’s manual (Ligation Protocol with T4 DNA Ligase, NEB). Recombinant plasmids were transformed into *E. coli* DH5α (NEB) by heat shock and selected by kanamycin- and ampicillin-containing LB plates at 37 °C overnight. Sequences of plasmid inserts of selected clones were verified by Sanger sequencing (Microsynth SeqLab).

### dsRNA synthesis for RNAi

We synthesized dsRNA of the neomycin resistance (*neoR*) gene from a 420 bp fragment of pJC53.2, which was previously amplified by PCR using gene-specific primers containing the T7 promotor sequence (Q5 High-Fidelity Polymerase, primers: Neomycin_T7_fw 5′-TAA TAC GAC TCA CTA TAG GGA GA G TGG AGA GGC TAT TCG GCT-3′ and Neomycin_T7_rev, 5′-TAA TAC GAC TCA CTA TAG GGA GA C ATC CTG ATC GAC AAG ACC G-3′; underlined is the T7 sequence). Previously amplified PCR products from templates using primers with 5′-T7-promoter overhangs (Q5 High-Fidelity Polymerase, NEB; T7_extended primer specific for pJC53.2 T7-promoter sequence 5’-CCT AAT ACG ACT CAC TAT AGG GAG-3’) served as templates for dsRNA synthesis. We synthesized dsRNAs of the *ampR* gene and the GFP gene from 493 to 529 bp long PCR products, respectively, derived from recombinant pJC53.2 (see 2.3., Supplementary Table S1). Amplification was achieved using primers against the T7-promoter sequence, as described above.

Approximately 0.5 μg PCR product was used for an in vitro transcription reaction^30^ with an in-house produced T7 RNA polymerase, expressed as described elsewhere^29^. The reaction mixture contained 10 µL transcription buffer (10x), 20 µL rNTP mix (NEB), 10 µg T7 RNA polymerase (expression vector kindly provided by Jim Collins), 1 µL inorganic pyrophosphatase (NEB), added with DEPC-treated water to 100 μl total volume. The reaction was carried out for approximately 16 h at 37 °C, followed by DNase I treatment (10 U, NEB, 37 °C) for 30 min. RNA was precipitated using 7.5 M LiCl_2_ and cleaned from the reaction mixture. Afterwards, the RNA was resuspended in DEPC-treated water and incubated for 3 min at 72 °C. Subsequently, the dsRNA concentration was determined by photometric measurement using the BioSpectrometer Basic (Eppendorf) and the synthesis of dsRNA confirmed by agarose gel electrophoresis. For RNAi, dsRNA was used in concentrations of 30 μg mL^−1^.

### In vitro culture and RNAi of *S. mansoni*

Adult *S. mansoni* were derived from hamsters by perfusion. Subsequently, we cultured the worms in M199(3+) (Gibco, supplemented with 1% (v/v) antibiotic–antimycotic solution (CCPro), 1% (v/v) HEPES buffer (Carl Roth, pH 7.4), and 10% (v/v) fetal calf serum (Sigma-Aldrich) in a CO_2_ incubator Galaxy S+ (RS Biotech; Germany) at 37 °C and 5% CO_2_. Adult worms adapted for one day to the in vitro culture conditions before dsRNA treatment started. For each of the three biological replicates, 10 *S. mansoni* couples obtained from one hamster were transferred in one well of a 6-well plate (Greiner bio-one), which was filled with 3 mL prewarmed (37 °C) M199 (3+) medium. For RNAi experiments, dsRNA was added by soaking in a concentration of 30 µg mL^−1^. The parasites were cultured for three weeks, with medium and dsRNA renewed every 2–3 d. Viability of the worms was monitored regularly by microscopic observation, along with medium exchange, as described before^29^ (scoring system: 0 = total absence of movement, 1 = only gut movements or occasional movement of head and tail, 2 = reduced motility, 3 = normal activity and 4 = hyperactivity). Furthermore, attachment of worms to the petri dish, pairing status (either paired or separated), and oviposition were determined next to morphological changes. Scoring was done with an inverse laboratory microscope (DM IL LED, Leica Microsystems). At the end of the treatment and monitoring period, 0.25% (w/v) ethyl 3-aminobenzoate methanesulfonate (Sigma-Aldrich) was used to separate couples^30^. Male and female worms were separately collected, washed with PBS and fixed for confocal laser scanning microscopy (CLSM)^31^, or the worms were frozen in DNA/RNA protection buffer for RNA isolation (Monarch Total RNA Miniprep Kit, NEB).

### Identification of potential RNAi off-target gene transcripts of non-schistosomal dsRNAs

In order to evaluate the potential knockdown effects of the selected dsRNAs on the expression of off-target genes, we utilized the software tool si-Fi^27^. The search was carried out using full-length transcript sequences obtained from the *S. mansoni* genome versions 7 (PREJA36577; WBPS version 15) and version 10 (PREJA36577; WBPS Version 18) available at the database WormBase ParaSite (https://parasite.wormbase.org/ftp.htm). Default parameters were used for si-Fi with a siRNA size of 17 nt and allowing one mismatch. Furthermore, dsRNA stability indicated by the Δ*H*-value^32,34^ was calculated by Oligo Calc: Oligonucleotide Properties Calculator^33^ applying the default setup for dsRNA.

### RNA isolation, cDNA synthesis, and quantitative RT-PCR analyses

RNA isolation of female and male *S. mansoni* was achieved using the Monarch Total RNA Miniprep Kit (NEB) following the manufacturer’s instructions. RNA was eluted in 50 μl DEPC-treated water. Subsequently, concentration and integrity of the total RNA were analyzed by electropherogram analysis (2100 Bioanalyzer instrument; Agilent Technologies) in combination with the RNA 6000 Nano Kit. cDNA synthesis for each RNA sample was performed with 100 ng total RNA in one reaction with the QuantiTect Reverse Transcription Kit (Qiagen). The final reaction mixture was incubated at 42 °C for 30 min and the reaction stopped at 95 °C for 3 min.

The reaction mixture for RT-qPCR consisted of 10 µL 2 × Quanta mix (Qiagen), 0.8 µL specific primer mix (forward and reverse primer, each 10 µM), 5 µL template cDNA and 4.2 µL PCR grade water (Carl Roth), which was established as standard approach earlier^35^. The cycling conditions were as follows: initial denaturation 95 °C for 3 min, followed by 45 cycles of DNA denaturation at 95 °C for 10 s, primer annealing at 60 °C for 15 s and elongation at 72 °C for 20 s. Amplification was followed by a melt curve analysis at 60–95 °C with stepwise increase of 1 °C for 20 s each cycle. Primers for RT-qPCR were designed targeting transcripts of predicted off-target genes (Supplementary Table S2). Primer efficiencies were determined as described before^34,35^. A primer pair was accepted for use, if the resulting efficiency value ranged between 0.9 and 1.1, and no primer dimer or nonspecific PCR products were detected after melt curve analysis. The transcript levels of genes of interest were determined by application of the 2^−ΔΔCt^ method^34^. Expression levels in control worms (incubated in medium supplemented by DEPC-treated water) were used for normalization, and Sm*letm-1* (SmLETM_qPCR2_fw CGT GGA ATG CGT TCA GTT GG and SmLETM_qPCR2_rev GAA GCT GAT GGA GGT AAT TGAG) as reference gene^35^.

### Carmine-red staining of *S. mansoni* and confocal laser scanning microscopy

Staining was carried out as described elsewhere^31^. In short, worms were fixed for 24 h in AFA-fixative (66.5% (v/v) ethanol, 1.1% (v/v) paraformaldehyde, 2% (v/v) glacial acetic acid in water). For staining, the worms were transferred to netwell inserts (Science Service) fitted into 12-well plates and incubated for 30 min in carmine-red solution (CertistainH, Merck). For destaining, the worms were incubated several times in acidic ethanol (70% (v/v), ethanol, 2.5% (v/v), and hydrochloric acid (Carl Roth) for 5–10 min. Next, the worms were dehydrated in 80%, 90%, and 100% ethanol for 5 min each, before mounting in Canada balsam (Sigma-Aldrich)^36^. For microscopy, a TCS SP5 vis confocal laser scanning microscope (CLSM; Leica Microsystems) was used. CLSM images were acquired using a 488 nm He/Ne laser and a 470 nm long-pass filter in reflection mode. Furthermore, background signals and optical section thickness were defined by setting the pinhole size to airy unit 1^37^.

### Statistics

Statistics were carried out using the GraphPad Prism V.8 software (GraphPad Software, San Diego, USA) applying the non-parametric Mann–Whitney t-test for non-normally distributed data. Statistical differences with *p* < 0.05 were considered significant.

### Ethics declaration and approval for animal experiments

All experiments involving hamsters have been performed in accordance with the European Convention for the Protection of Vertebrate Animals used for Experimental and other Scientific Purposes (ETS No 123; revised Appendix A) and have been approved by the Regional Council (Regierungspraesidium) Giessen (V54‐19c20 15h 02 GI18/10).

## Results

### Bioinformatics analysis of *non-schistosomal dsRNAs*

Aim of the following experiments was to evaluate three selected non-target dsRNAs for their potential to serve as reliable controls for RNAi experiments. We chose non-helminthic sequences of the plasmid-encoded neomycin resistance gene (*neoR*), ampicillin resistance gene (*ampR*), and green fluorescent protein (*gfp*). Since many studies in schistosomes, including those seeking to characterize new targets for intervention strategies, focus on adults and their reproduction biology, we used *S. mansoni* couples as study object.

We designed the candidate dsRNAs to a commonly used length between 400 and 500 bp. As it is known that features like GC-content can influence the RNAi maschinery^38^, we assessed these selected parameters shown in Table 1. GC-content ranged between 44 and 47% for *ampR* and *gfp*, while 59% was observed for *neoR.* To investigate whether the candidate dsRNAs are prone to be fragmented into siRNAs with the potential to bind to off-target sequences, we first made use of the si-Fi software^27^. This tool dissects dsRNA sequences into potential siRNA sequences in silico and predicts their target mRNA sequences. For the *neoR*, *ampR*, and *gfp* dsRNAs, si-Fi predicted 6, 41, and 116 potential off-target genes in *S. mansoni,* respectively (Table 1, Supplementary Table S3). The frequency of siRNA-binding to these genes ranged from 1 (for most of the detected transcripts) to 7 (Smp_130480; *gfp* dsRNA). Furthermore, the Δ*H*-value as an indicator of structural stability of dsRNA was calculated (Table 1). The dsRNAs *ampR* and *gfp* showed highest Δ*H-*values at a comparative level. In contrast, a much lower Δ*H-*value was calculated for the dsRNA *neoR*.

**Table 1:** Parameters of the selected non-schistosomal dsRNAs.

Sequence	Length (bp)	GC-content (%)	Stability Δ*H* kcal/mol	Potential targets
*ampR*	493	47	5229	41
*gfp*	529	44	5526	116
*neoR*	420	59	4768	6

To investigate possible knockdown effects of *neoR*, *ampR*, and *gfp* dsRNAs, worm morphology, vitality, and egg production were monitored next. In addition, RNA was isolated from dsRNA-treated worms for RT-qPCR analyses to investigate transcript levels of selected, putative off-target genes.

### Phenotypic analyses of dsRNA-treated *S. mansoni*

**Figure 1:**
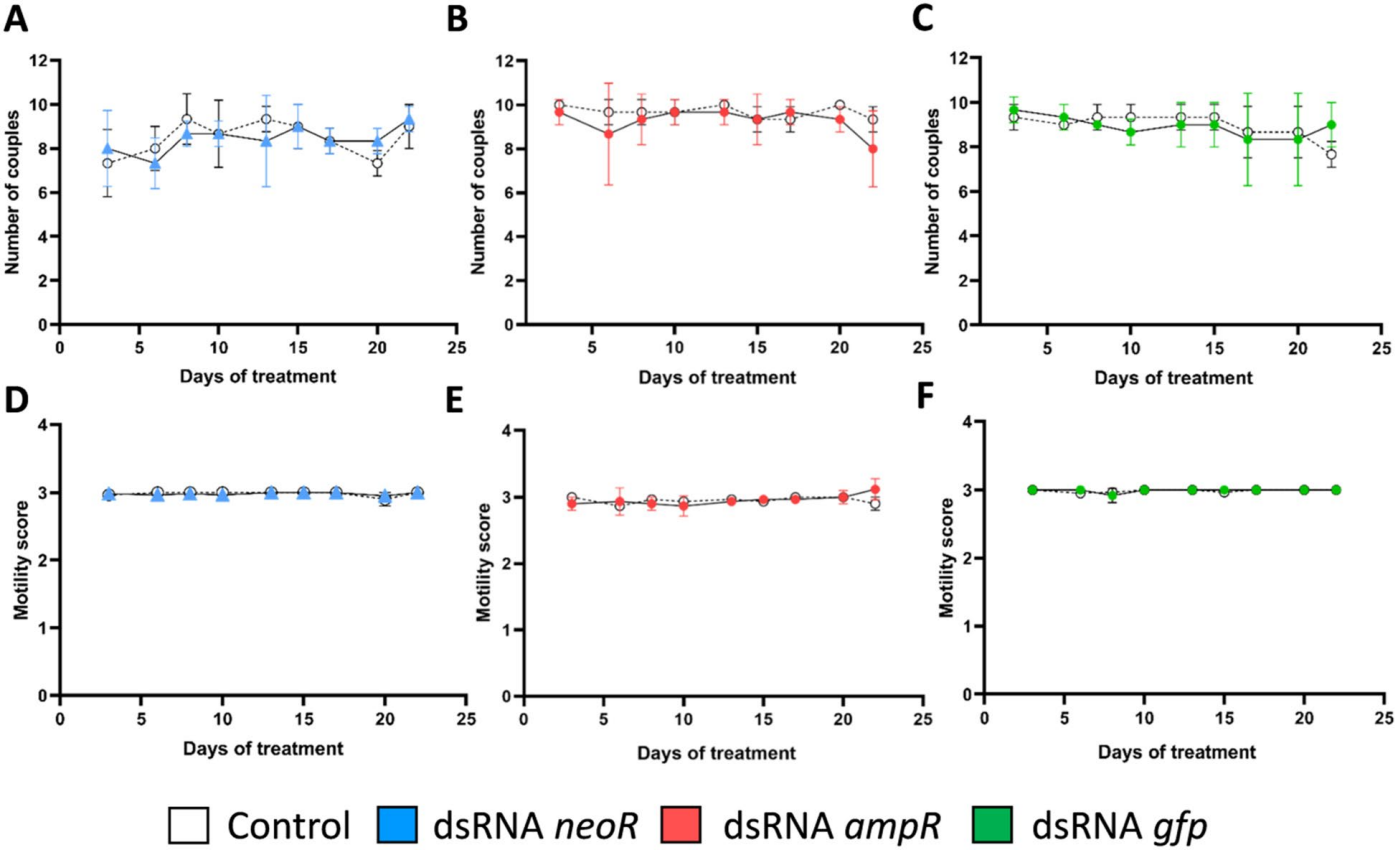
dsRNAs of non-schistosomal origin showed no effects on pairing stability and motility of *S. mansoni* couples in vitro. Couples were treated with 30 µg/mL dsRNA every 2–3 days over a period of 22 days. Each dsRNA was directed against a different target gene: blue (*neoR*), red (*ampR*), and green (*gfp*). Couples treated with DEPC-H_2_O instead of dsRNA served as control (white). Compared to the control, no effects of the different dsRNA treatments on the number of couples (**A–C**) and motility (**D–F**) as parameters for pairing-stability and vitality, respectively, were observed during the experimental period. *ampR* ampicillin resistance gene, *gfp* green fluorescent protein gene, *neoR* neomycin resistance gene; n = 3.

Adult female schistosomes acquire sexual maturity through a constant mating contact with male partners, which is the prerequisite for egg production^39,40^. Several studies have shown that gonad differentiation in the female is reversible since vitellarium and ovary dedifferentiate after separation of the female from the male^40–43^. To investigate whether the physiology of *S. mansoni* couples during a typical in vitro study may be influenced by off-target activity of *neoR*, *ampR*, or *gfp* dsRNAs, their individual effects on motility and pairing-stability were monitored during a dsRNA-treatment period of 22 d (Fig. 1A–C). Couples treated with DEPC water instead of dsRNA served as controls. Compared to the control, no significant differences in the number of couples were observed for any of the three tested dsRNAs. In addition, worm motility was not affected during the observation period (Fig. 1D–F). Motility values averaged 3 throughout the whole experimental period for all groups. In addition, no dsRNA-dependent effect on the attachment capacity of the worms was observed (data not shown). In summary, we observed no effect of *neoR*, *gfp*, or *ampR* dsRNAs on pairing stability and motility.

### Control dsRNA candidates take no effect on egg formation in *S. mansoni*

**Figure 2:**
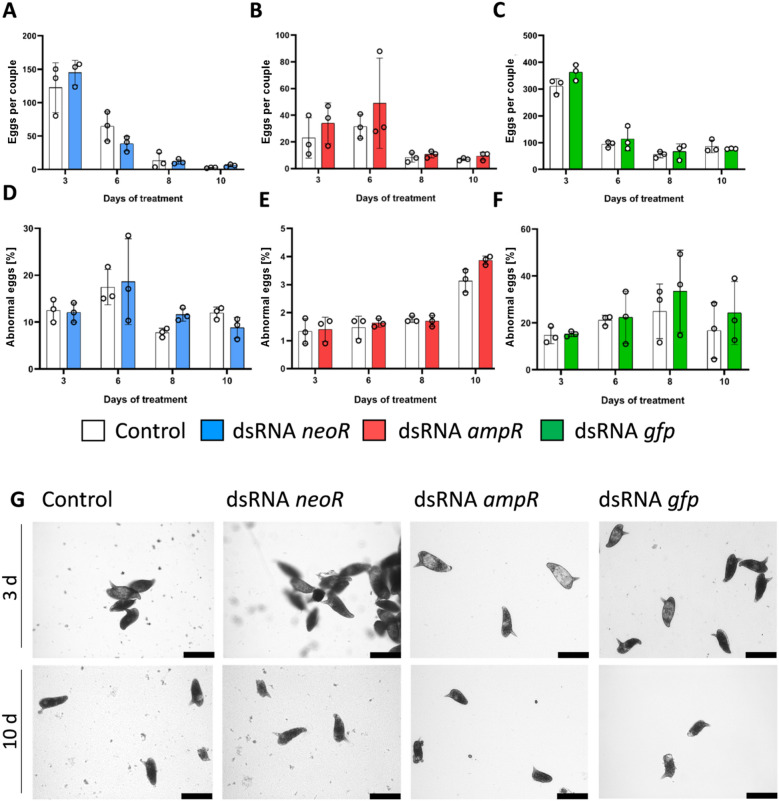
Non-schistosomal dsRNAs showed no quantitative or qualitative effect on egg production of *S. mansoni* couples in vitro. In vitro-generated eggs of dsRNA-treated *S. mansoni* couples were monitored during a 10 d observation period in culture. Couples were treated with 30 µg/mL of the respective dsRNA (blue, *neoR*; red, *ampR*; green, *gfp*), which was added every 2–3 days for a period of 22 d. DEPC-H_2_O-incubated, but otherwise untreated couples served as control (white). To determine the effects of different dsRNAs on the egg numbers, the total number of in vitro-generated eggs, normalized to the overall number of couples, was calculated for each experimental group (**A–C**). In addition, the number of malformed eggs normalized to the number of couples was determined for each experimental group (**D–F**). No morphological effects of the tested dsRNAs were observed in comparison to the control. In (**G**), the morphology of in vitro-generated eggs after 3 d and 10 d is shown (bright-field microscopy). Compared to the controls, no morphological effects following dsRNA treatment were observed for either time point. Scale bars: 100 µm. *ampR* ampicillin resistance gene, *neoR* neomycin resistance gene, *gfp* green fluorescent protein gene; n = 3.

Number and synthesis of normal eggs are suitable indicators of the fitness of schistosome couples in vitro. Previous experiences of many labs working with adult schistosomes in vitro have shown a decline of the total number of eggs per (non-treated) couple after 3 to 6 days of culture^44–48^. We observed similar tendencies in our experiments. Nonetheless, in our hands egg production occurred during a 10 d period in vitro. Comparative analyses between all experimental groups showed no significant differences in egg production (Fig. 2A–C). Also, the proportion of malformed eggs in relation to the total number of eggs per couple showed no statistically significant difference (Fig. 2D–F). Of note, we observed differences in the quantity and quality of eggs between the different biological replicates used.

Furthermore, the morphology of eggs produced until day 3, and between days 8 and 10 was analyzed (Fig. 2G). Eggs of the day 3 groups showed the expected stages I–III of egg development according to Jurberg's staging system^49^. However, eggs laid at the late time points displayed developmental stages I and II but not stage III. Furthermore, we observed an increase of abnormal eggs for all groups over the time in culture (Fig. 2D–F). This was expected as it has been previously described for in vitro-generated schistosome eggs^44^^.^

### CLSM analyses of dsRNA-treated *S. mansoni*

**Figure 3:**
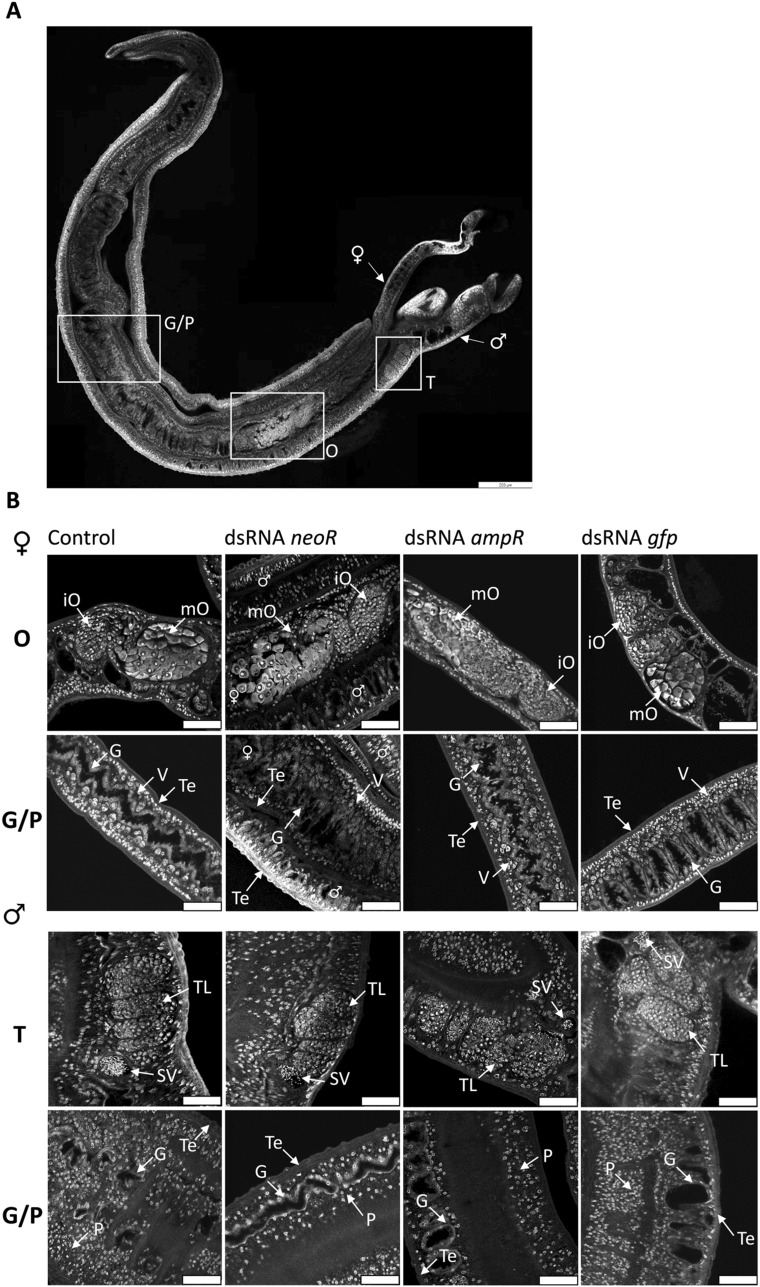
Non-schistosomal dsRNAs caused no morphological changes in *S. mansoni* couples. Couples were treated with 30 µg/mL dsRNA every 2–3 days over a period of 22 days, each encoding different dsRNA sequences (*neoR*, *ampR,* and *gfp*). Couples treated with DEPC-H_2_O instead of dsRNA served as control. After the treatment period, couples were analyzed by CLSM. (**A**) Representative image showing an overview of the morphology of a *S. mansoni* couple. Testes (T), ovary (O), gut (G), and parenchyma (P) are framed. Scale bar: 200 µm. (**B**) Representative pictures of the female and male reproduction organs (ovaries (O) and testes (T)), as well as gut and parenchyma (G/P) are shown for the control and dsRNA-treated worms, respectively. No obvious morphological differences were observed between the different groups. Scale bars: 50 µm. *ampR* ampicillin resistance gene, *gfp* green fluorescent protein gene, *neoR* neomycin resistance gene, *iO* immature ovary, *G* gastrodermis, *mO* mature ovary, *P* parenchyma, *T* testes, *Te* tegument, *TL* testicular lobe, *SV* seminal vesicle, *O* ovary, *V* vitellarium; n = 3 (5 couples/n).

To examine potential effects of dsRNA treatment on the tissue integrity of the reproductive organs, the gastrodermis, and the parenchyma, treated worms were analyzed by CLSM. For each condition, we analyzed 15 couples (5 couples per biological replicate) (Fig. 3). We detected no obvious structural differences between the control and the dsRNA-treated worms after 22 d. In females of all groups, the development of the ovary was unaffected and showed the typical division into an anterior and posterior part containing immature and mature oocytes, respectively (Fig. 3B)^36^. In addition, the vitellaria of dsRNA-treated worms showed no difference to the control. In males, neither the structure of testicular lobes and seminal vesicle nor the occurrence of spermatogonia appeared to be affected by the dsRNA treatments (Fig. 3B)^36^. Subsequently, we analyzed the integrity of other tissues, the tegument, the parenchyma, and the gut with emphasis on the gastrodermis. Again, we found no phenotypic differences between the control and the dsRNA-treatment groups (Fig. 3B). In summary, no obvious phenotypic effects on schistosome tissues were observed by CLSM.

### Validation of dsRNA-induced effects on expression levels of predicted off-target mRNAs

Next, we studied potential effects of the candidate dsRNAs on the transcript levels of predicted off-targeted genes using RT-qPCR. To this end, genes were selected that showed the highest number of off-target dsRNA hits (> 3) for a certain dsRNA, and which were shown before to be transcribed in paired adult schistosomes^50,51^. For *neoR* dsRNA, the transcript levels of all six potential off-target genes were analyzed as there were no more than 2 siRNA hits for each of these genes.

**Figure 4:**
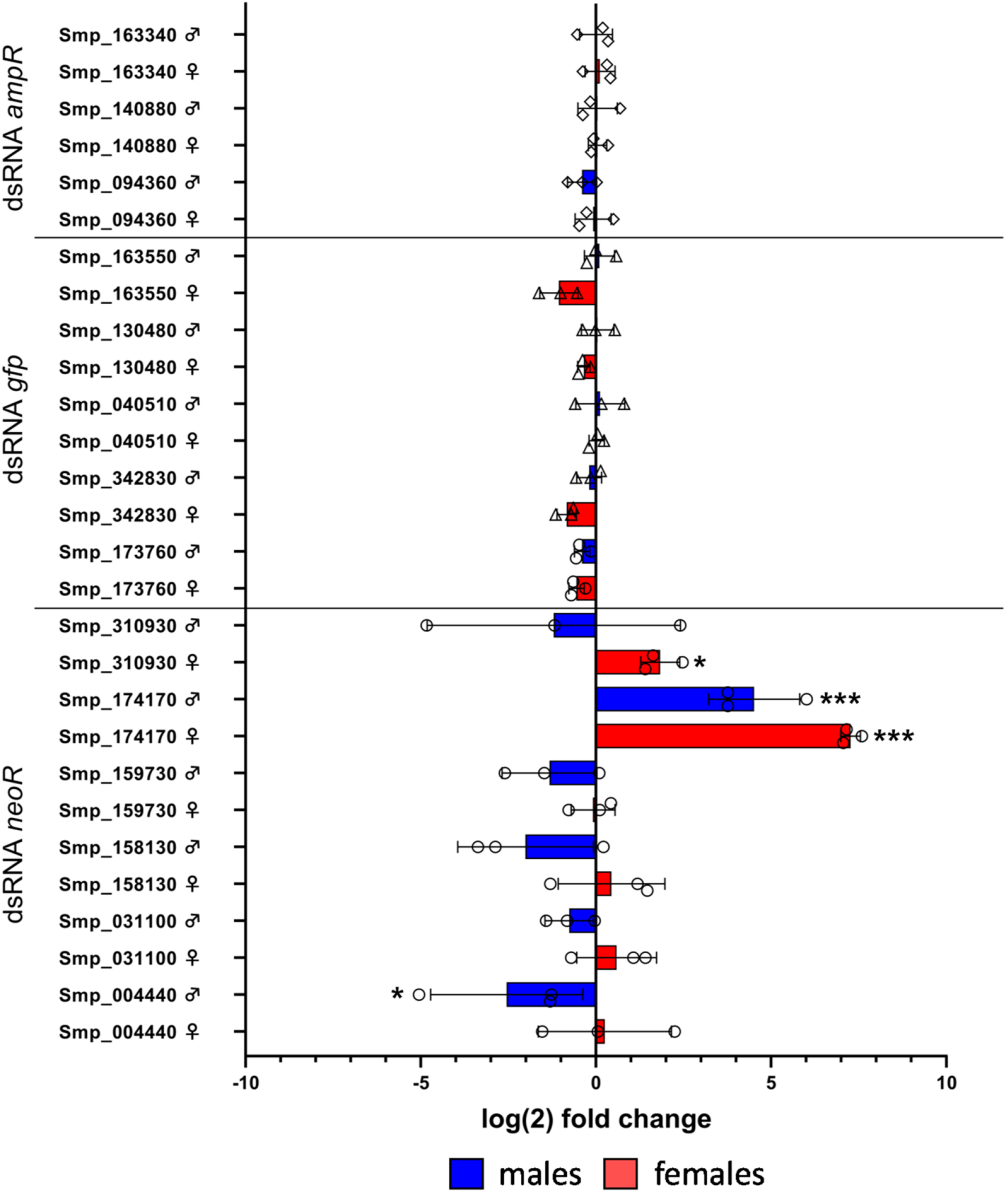
Non-schistosomal dsRNAs influenced transcript levels of predicted off-target genes. The effects of non-schistosomal dsRNAs (*neoR, ampR* and *gfp*) on the transcript levels of potential off-target genes, as predicted by si-Fi^27^, was analyzed by RT-qPCR. The logarithmic changes (log(2)-fold changes) of dsRNA-treated and control couples were determined after a treatment period of 22 days. To display potential sex-dependent effects, log(2)-fold changes were separately determined for females (red) and males (blue). We focused this analysis on the top-ranking genes predicted as off-targets of *neoR* (6), *gfp* (4), and *ampR* (3). Shown are the respective calculated mean values (bars), the individual biological replicates (circles, triangles, and diamonds), and the standard errors of the mean. The transcript levels of predicted off-target genes of couples treated with the dsRNA *ampR* were comparable to those of the respective controls. For g*fp* dsRNA-treated couples, a modest reduction of the transcript levels of Smp_163550 and Smp_342830 of female schistosomes was found. In the analysis of couples treated with the dsRNA *neoR*, strong changes in the transcript levels of potential off-target genes were detected for both sexes compared to controls, being highest for Smp_174170. *ampR* ampicillin resistance gene, *gfp* green fluorescent protein gene, *neoR* neomycin resistance gene; n = 3.

Compared to the control group, the log(2)-fold changes of the transcript levels of the *neoR* dsRNA-treatment group revealed a mixed picture (Fig. 4). Following treatment, especially the transcript levels of four of the six predicted off-target genes, Smp_00440, Smp_158130, Smp_159730, and Smp_310930, were reduced. This was observed for male schistosomes, whereas no changes of the transcript levels for these genes were observed in treated females. For Smp_174170, an increased transcript level was observed in the *neoR* dsRNA-treated group in both sexes. Here, a log(2)-fold change of 7.37+/− 0.22 was determined for males and a log(2)-fold change of 4.51+/− 1.06 for females.

Treatment with *gfp* dsRNA showed only marginal changes in the transcript-levels of selected off-target genes that were more pronounced in female schistosomes (Smp_163550: log(2)-fold change − 1.06+/− 0.45 and Smp_342830: log(2)-fold change − 0.84+/− 0.22). Four potential siRNA hits had been predicted for Smp_163550 and Smp_342830. No deviation from controls was detected for transcript levels of the remaining genes, despite high predicted off-target frequencies, such as for Smp_130480 (7) and Smp_040510 (5).

Finally, we detected no effects on the transcript levels of genes predicted as potential targets of *ampR* siRNAs. Transcript profile changes were marginal and averaged at a comparable level to the controls. Only a minimal decrease in the transcript abundance was detected in females for Smp_094360 (log(2)-fold change − 0.08+/− 0.42), which had been predicted to have six non-target dsRNA/ siRNA hits. Smp_094360 showed the highest number of off-target hits (6), while for Smp_140880 5 and for Smp_163340 4 hits had been predicted (Supplementary Table S3).

## Discussion

RNAi is a well-established technical approach for functional gene analyses in many organisms including parasites. Although frequently applied^52^, only one study exists for schistosomula of *S. mansoni* with GFP or mCherry nonspecific dsRNAs as controls, showing transcriptional changes of several genes with each control^18^. To our knowledge, however, no further study exists that has systematically addressed the question of suitable RNAi controls using non-parasitic dsRNA for RNAi experiments in schistosome adults, other multicellular parasites, or free-living worms. As exemplified for the parasite *S. mansoni*, we selected three non-schistosome dsRNAs, *neoR*, *ampR*, and *gfp,* and investigated their suitability as control dsRNAs. For monitoring potential effects caused by these candidate dsRNAs, we investigated their impact on physiological parameters like pairing stability, motility, and egg production as well as morphological aspects. Furthermore, we predicted off-target effects by a bioinformatics approach using si-Fi^27^, a software tool that dissects dsRNA in silico into hypothetical siRNA sequences and subsequently maps these against potential target genes using the BOWTIE algorithm. Consequently, we performed RT-qPCRs to determine transcript levels of the predicted off-target genes to get hints about unwanted effects on the expression of such genes in *S. mansoni*.

At the physiological level, we observed no effects of all candidate dsRNAs on pairing stability, motility, and egg production. We noted a high variability of the number of eggs produced by couples among the different datasets, but not within individual datasets. This phenomenon has been observed before^44^ and can be explained by a host influence (different hamster populations) or genetic variation among the various worm populations represented by the biological replicates of *S. mansoni* used in this study. Since no isogenic hamster strains exist, natural variability of the host environment cannot be avoided. Beyond that, although culture conditions for schistosomes have remarkably improved over the last decades^44,53–55^, today’s culture protocols still do not allow long-term egg production. In most media formulations used, egg production declines over time, depending on the media between one to three weeks^44–48^. Even though this poses a general challenge, our results indicate no negative effect of the used dsRNAs on egg production under our standard culture conditions. We next analyzed the morphological structure of important schistosome organs, such as the gonads of both sexes, by CLSM. Again, we found no differences between untreated controls and dsRNA-treated worms, a finding that corresponds to the normal egg production observed in the treatment groups.

The absence of physiological and morphological alterations cannot exclude possible off-target effects of non-schistosome dsRNAs at the molecular level. Potential effects at the transcript level might be caused by either siRNAs, which accidentally show sequence homologies to schistosome transcripts, or they could originate from a more general dysregulation of the RISC complex. It has been shown that dsRNA used in an RNAi experiment can lead to a competition of exogenous and endogenous regulatory RNAs^56–58^. A high concentration of non-target dsRNA in schistosomula was shown to induce abnormal phenotypes or even the death of the parasite^23,26^. To address this point, we measured the transcript levels of genes predicted to be off-targets of siRNAs, which were potentially generated by the non-schistosome dsRNAs used in this study. This approach uncovered a variable degree of off-target effects by the different non-schistosome dsRNAs, helping us to narrow down the most suitable candidate dsRNA control.

Transcript levels of the predicted (and for analysis selected) off-target genes of *neoR* dsRNA were highly affected. While some of these genes showed a strong increase in transcript abundance, which was unexpected, the transcript levels of other genes were reduced, indicating an off-target knockdown. Prominent upregulation was observed especially for the transcripts of Smp_174170, which has been annotated as homeobox protein Meis3-A^51^. The unexpected increase in its transcript level might hint at a natural regulation of the expression of this gene by the parasites regulatory RNA machinery. Previous studies have provided evidence that small RNAs may have the potential to even activate gene expression by targeting gene regulatory sequences, including promoters^58–60^_._ With respect to Smp_174170, a previous meta-analysis indicated that transcript levels of this gene peaked in the schistosomula stage, whereas transcript abundance was low in adult *S. mansoni* in both sexes^51^. Furthermore, single-cell transcript analysis of adult schistosomes showed its neuron-specific transcript occurrence^61^. In vertebrates, Meis3-A orthologs can function as transcription activators during early neuronal development^62,63^. As homeobox (HOX) gene, Smp_174170 might be involved in neuronal development during the transition between different larval stages^64,65^ and neurotransmitter maintenance^4^. Due to the absence of a clear physiological and morphological phenotype in adults in our study, we suspect that phenotypic alteration may more likely occur at the schistosomula stage. Besides Smp_174170, *neoR* dsRNA-treated worms revealed a dysregulation of transcripts of all other predicted off-target genes including the putative serine/threonine-protein kinase PAK 3 (Smp_159730)^51^. Serine/threonine protein kinases play important roles in a variety of different signalling pathways including cytoskeleton regulation, cell migration, and cell-cycle regulation^66–68^. Furthermore, we observed the downregulation of Smp_004440 in males, a putative member of the neurexophilin and PC-esterase (NXPE) family of neuropeptide-like glycoproteins. Since the Smp_004440 transcript level increases from the 3 h schistosomulum stage to 28 day-old juveniles^51^, this gene may be important for the growth phase of schistosomes in the final host and not so much for the maintenance of the adult stage, especially of the males, which may explain the absence of a phenotype. As these regulations were highly variable, we reason that this might indicate a high saturated RISC and connected dysregulation of transcription rather that specific mediated degradation, as it was shown for *Paramecium*^56^. As we used similar concentration for all dsRNAs, this might hint at differing sequence-specific affinities of the RISC. Furthermore, the notable sequence differences between *neoR* and the sequences of the other two genes, which showed lower GC-content and higher Δ*H*-values compared to *neoR*, might hint at a different influence of these sequence features on RNAi efficacy. The influence of GC content or thermodynamic stability is a described phenomenon^29^. Other known aspects are the influences of sequence cleavage preference in dsRNA processing by dicer, which was described in *Paramecium,* moths, or grasshoppers^69,70^. As to date, there is no detailed characterization of the RNAi machinery in schistosomes, thus further work is needed to determine the influence of sequence composition of dsRNAs on RNAi efficacy in this parasite. All in all, this data set strongly indicates that *neoR* dsRNA is not suitable as a control for RNAi experiments.

For *gfp* dsRNA-treated worms, a decrease in transcripts for two genes was noticed, but only in female schistosomes. Target genes encoded for Smp_163550 (hypothetical protein) and Smp_342830 (putative CD109 antigen)^51^. In humans, CD109 plays a role in osteoclast proliferation^71^ and in the development of squamous carcinoma when it is overexpressed^15^, which could indicate a potential influence of *gfp* dsRNA treatment in developmental processes and has to be taken into consideration when using this control. Of note, in the honey bee, Nunes et al.^57^ found tremendous effects of the (in this system) commonly used *gfp* dsRNA as RNAi control. In microarray experiments, effects on gene expression were remarkably high, 1,400 genes were shown to be deregulated. Furthermore, *gfp* dsRNA influenced pigmentation and developmental timing of the honey bee.

For *ampR* dsRNA-treated worms, finally, with the exception of Smp_094360, no effects on the transcript levels of predicted off-target genes were found. Smp_094360 transcript levels appeared to be slightly reduced. This gene is predicted to code for a remodelling and spacing factor 1, and its transcript level declines from the schistosomulum stage to the adult stage^51^, in which it may be of minor importance.

Comparing all parameters used in this study to monitor off-target effects of three non-schistosome dsRNAs upon their application to adult *S. mansoni *in vitro, we conclude that the *ampR* dsRNA may be best suited as control for RNAi experiments. As the *neoR* and *gfp* dsRNAs, *ampR* dsRNA caused no physiological or morphological phenotype in in vitro-culture experiments performed for a three week period and with a concentration of 30 µg/mL dsRNA in each case. Compared to *neoR* and *gfp* dsRNAs, however, the *ampR* dsRNA exhibited—with one negligible exception—no influence on the transcript levels of the 6 most likely predicted off-target genes out of 41 total. One additional finding of our study was the sex-dependent effect of especially *neoR* dsRNA on some off-target gene transcripts, as it has been demonstrated for Smp_159730, Smp_158130 and Smp_004440. This indicated that not only the dsRNA sequence and its target transcript, but also the sex might eventually impact RNAi efficiencies and downstream analyses. Recently, the genome version used in this study received a major update from version 7 (V7) to version 10 (V10), improving on several aspects compared to the previous assembly. In order to validate our results, we performed the in silico off-target prediction on transcript sequences of the new version. Compared to our results with V7, in V10 we detected an increase in predicted targets for *neoR* (from 6 to 11 predicted targets) and *ampR* (from 41 to 49 predicted targets) dsRNAs. The most notable changes were present in the numbers of predicted off-targets for the *gfp* dsRNA decreasing from 116 predicted targets to 70, which resulted from the fusion of different Smp-numbers to single genes, the reduction of the number of splice variants, as well as a notable change in top hit genes. The changes are caused by a combination of factors like the change of average transcript length from 2794 bp in V7 to 3600 bp in V10, which increases the probability of siRNA mapping as well as a change in gene prediction by applying different gene models. These changes affected the annotations of several transcript start sites and/or the numbers of splice variants of certain genes. Nonetheless, we noted that the *ampR* targets remained mostly stable in both versions, further emphasizing this sequence as a valuable dsRNA control.

In our study, we monitored the change in gene expression after an experimental period of 22 days. As transcription is a highly dynamic process, a limitation of our study is the lack of transcript profile determination at earlier time points. Nonetheless, the conclusions are valid for the time point chosen for this work, and more research could illuminate if the responses are unstable over time. Therefore, we emphasize the importance of a careful experimental design for RNAi experiments, especially when using novel dsRNAs for a target gene of interest in a specific physiological or developmental context—such as the parasite stage and sex, in which the RNAi experiment will be performed. The presented data can serve as a valuable basis for the design of RNAi experiments with schistosomes or other worms and provides guidelines for the choice of non-schistosome dsRNA as negative control.

## Supplementary Information

Supplementary Information 1.

Supplementary Information 2.

Supplementary Information 3.

Supplementary Information 4.

## Data Availability

To enhance reproducibility of this study, we included all relevant raw-data in Supplementary Table S4.
