# Supplementary material for: Defining an optimal control for RNAi experiments with adult Schistosoma mansoni
Source: Sci Rep. 2023 Jun 16;13:9766. doi: 10.1038/s41598-023-36826-6 (PMC10276032; doi:10.1038/s41598-023-36826-6)
Supplement: Supplementary file 1 — Supplementary Information 1. [file 41598_2023_36826_MOESM1_ESM.docx]

**Supplementary Table S1**. Compilation of sequences used as template for dsRNA synthesis and primers used for amplification of the fragments, respectively

**Ampicillin resistance gene**

**dsRNA template**

GAGTATTCAACATTTCCGTGTCGCCCTTATTCCCTTTTTTGCGGCATTTTGCCTTCCTGTTTTTGCTCACCCAGAAACGCTGGTGAAAGTAAAAGATGCTGAAGATCAGTTGGGTGCACGAGTGGGTTACATCGAACTGGATCTCAACAGCGGTAAGATCCTTGAGAGTTTTCGCCCCGAAGAACGTTTTCCAATGATGAGCACTTTTAAAGTTCTGCTATGTGGCGCGGTATTATCCCGTATTGACGCCGGGCAAGAGCAACTCGGTCGCCGCATACACTATTCTCAGAATGACTTGGTTGAGTACTCACCAGTCACAGAAAAGCATCTTACGGATGGCATGACAGTAAGAGAATTATGCAGTGCTGCCATAACCATGAGTGATAACACTGCGGCCAACTTACTTCTGACAACGATCGGAGGACCGAAGGAGCTAACCGCTTTTTTGCACAACATGGGGGATCATGTAACTCGCCTTGATCGTTGGGAACCG

**Amplification primers**

Ampicillin fw GAG TAT TCA ACA TTT CCG TGT CGC

Ampicillin rev CGG TTC CCA ACG ATC AAG GC

**GFP**

**dsRNA template**

GCAACATACGGAAAACTTACCCTTAAATTTATTTGCACTACTGGAAAACTACCTGTTCCTTGGCCAACACTTGTCACTACTTTCTGTTATGGTGTACAATGCTTTTCAAGATACCCAGATCATATGAAGCGGCACGACTTCTTCAAGAGCGCCATGCCTGAGGGATACGTACAGGAGAGGACCATCTCTTTCAAGGACGACGGGAACTACAAGACACGTGCTGAAGTCAAGTTTGAGGGAGACACCCTCGTCAACAGGATCGAGCTTAAGGGAATCGATTTCAAGGAGGACGGAAACATCCTCGGCCACAAGTTGGAATACAACTACAACTCCCACAACGTATACATCACTGCAGACAAACAAAAGAATGGAATCAAAGCTAACTTCAAAATTAGACACAACATTGAAGATGGAAGCGTTCAACTAGCAGACCATTATCAACAAAATACTCCAATTGGCGATGGCCCTGTCCTTTTACCAGACAACCATTACCTGTCCACACAATCTGCCCTTTCGAAAGATCCCAACG

**Amplification primers**

GFP-RNAi fw GCA ACA TAC GGA AAA CTT ACC C

GFP-RNAi rev CGT TGG GAT CTT TCG AAA GGG

**Neomycin resistance gene**

**dsRNA template**

GTGGAGAGGCTATTCGGCTATGACTGGGCACAACAGACAATCGGCTGCTCTGATGCCGCCGTGTTCCGGCTGTCAGCGCAGGGGCGCCCGGTTCTTTTTGTCAAGACCGACCTGTCCGGTGCCCTGAATGAACTGCAAGACGAGGCAGCGCGGCTATCGTGGCTGGCCACGACGGGCGTTCCTTGCGCAGCTGTGCTCGACGTTGTCACTGAAGCGGGAAGGGACTGGCTGCTATTGGGCGAAGTGCCGGGGCAGGATCTCCTGTCATCTCACCTTGCTCCTGCCGAGAAAGTATCCATCATGGCTGATGCAATGCGGCGGCTGCATACGCTTGATCCGGCTACCTGCCCATTCGACCACCAAGCGAAACATCGCATCGAGCGAGCACGTACTCGGATGGAAGCCGGTCTTGTCGATCAG

**Amplification primers**

Neomycin fw TAA TAC GAC TCA CTA TAG GGA GA G TGG AGA GGC TAT TCG GCT

Neomycin rev TAA TAC GAC TCA CTA TAG GGA GA C ATC CTG ATC GAC AAG ACC
