# Supplementary material for: Defining an optimal control for RNAi experiments with adult Schistosoma mansoni
Source: Sci Rep. 2023 Jun 16;13:9766. doi: 10.1038/s41598-023-36826-6 (PMC10276032; doi:10.1038/s41598-023-36826-6)
Supplement: Supplementary file 2 — Supplementary Information 2. [file 41598_2023_36826_MOESM2_ESM.docx]

**Supplementary Table S2**. Compilation of the predicted off-target transcripts and primers used to perform RT-qPCR analysis.

| **dsRNA** | **Transcript** | **Description** | **Primer name** | **Primer Sequence** | **Efficency** |
| --- | --- | --- | --- | --- | --- |
| **dsRNA *neoR*** | **Smp_159730** | Protein kinase | Smp-159730_fw | GTT CGA GCA TTG TAT CTT ATC  GC | 2.0 |
|  |  |  | Smp-159730_rev | GTT TGG AAC ATC GGC ATA TG |  |
| **dsRNA *neoR*** | **Smp_004440** | N/A | Smp-004440_fw | CGT GGG TGG TGT ACG ATG | 2.0 |
|  |  |  | Smp-004440_rev | ATG CAT TCG TCT CGA CCA G |  |
| **dsRNA *neoR*** | **Smp_158130** | Ethanolamine-phosphate cytidylyltransferase | Smp-158130_fw | ACC AAC ATT AGA GGG CTT AGA  CC | 2.0 |
|  |  |  | Smp-158130_fw | GCC AAC TGC ATT TCT TTA CTC  AC |  |
| **dsRNA *neoR*** | **Smp_031100** | Putative liquid facets | Smp-031100_fw | CCA GCT TCT ACA AAT CCA TTC  AC | 2.0 |
|  |  |  | Smp-031100_rev | GAT AGA ACA TTG GATTAT TCG  TTG TG |  |
| **dsRNA *neoR*** | **Smp_310930** | AMP deaminase | Smp-310930_fw | TCA TTT AGT TAC TTG TTT CCT  GTT GG | 1.9 |
|  |  |  | Smp-310930_rev | CTA AAA AGT CAT TCA ACG GAT  TAC G |  |
| **dsRNA *neoR*** | **Smp_174170** | Putative homeobox protein Meis3-A | Smp-174170_fw | CCA TCG GAA GAA CAA AAG AAA  C | 2.0 |
|  |  |  | Smp-174170_rev | CTT GAG CTG CTC TGG TAT AAG  CT |  |
| **dsRNA *gfp*** | **Smp_342830** | n/a | Smp-342830_fw | TGC ATG TGG AAG ATG GGT  TGG | 1.88 |
|  |  |  | Smp-342830_rev | CTA GAA TTG GCA CGT GCA TAT  CG |  |
| **dsRNA *gfp*** | **Smp_040510** | Putative alpha-mannoside beta-1,6-n-acetylglucosaminyltransferase | Smp-040510_fw | CCA GTC AAC ATC CGT ACA TAG  AAA ATC | 1.8 |
|  |  |  | Smp-040510_rev | TGT CAA ATA AGC ATT AAG ACG  TTC GAG |  |
| **dsRNA *gfp*** | **Smp_130480** | TBC1 domain family member 15, putative | Smp-130480_fw | ATT TCG GT TAC TTC CAA GGA  ATG AAT G | 1.88 |
|  |  |  | Smp-130480_rev | ACT GAA AGT CGG ATC AAC TAT  TTC AAT C |  |
| **dsRNA *gfp*** | **Smp_173760** | n/a | Smp-173760_fw | CCA CGT CAA CGA TTA TAT TGC  AAA TC | 1.88 |
|  |  |  | Smp-173760_rev | CGT CGT ATT TCA CAA GTA CTC  ACC |  |
| **dsRNA *gfp*** | **Smp_163550** | n/a | Smp-163550_fw | CAA TGC CAA ATC ACT ACA GTA  ATC | 1.88 |
|  |  |  | Smp-163550_rev | CTA ATC CCA TCG GTA AAT AAT  GTT C |  |
| **dsRNA *ampR*** | **Smp_094360** | putative Hepatitis B Virus X associated protein | Smp_094360_fw | TTC GAC ACG TGG AAA TAC TCG | 1.88 |
|  |  |  | Smp_094360_rev | TTC ACG ATA ACT AAC AAC AGT  TCG |  |
| **dsRNA *ampR*** | **Smp_140880** | Kruppel-like Factor | Smp_140880_fw | GTC AAG CCA TTT AAA GGC TCA | 1.99 |
|  |  |  | Smp_140880_rev | GCC TCG TTA ATT CAT CTG AAC  G |  |
| **dsRNA *ampR*** | **Smp_163340** | timeless | Smp_163340_fw | GGG ATG ATG CTG AAC GAA AGA | 1.86 |
|  |  |  | Smp_163340_rev | AGT TGC CAT TTG GGC TGT ATG |  |
